# Measurement of CD8^+^ and CD4^+^ T Cell Frequencies Specific for EBV LMP1 and LMP2a Using mRNA-Transfected DCs

**DOI:** 10.1371/journal.pone.0127899

**Published:** 2015-05-29

**Authors:** Dae-Hee Sohn, Hyun-Jung Sohn, Hyun-Joo Lee, Seon-Duk Lee, Sueon Kim, Seung-Joo Hyun, Hyun-Il Cho, Seok-Goo Cho, Suk-Kyeong Lee, Tai-Gyu Kim

**Affiliations:** 1 Department of Microbiology, College of Medicine, The Catholic University of Korea, Seoul, Korea; 2 Hematopoietic Stem Cell Bank, College of Medicine, The Catholic University of Korea, Seoul, Korea; 3 Cancer Research Institute, College of Medicine, The Catholic University of Korea, Seoul, Korea; 4 Department of Hematology, Department of Internal medicine, College of Medicine, The Catholic University of Korea, Seoul, Korea; 5 Research Institute of Immunobiology, Department of Medical Lifescience, College of Medicine, The Catholic University of Korea, Seoul, Korea; The University of North Carolina at Chapel Hill, UNITED STATES

## Abstract

An EBV-specific cellular immune response is associated with the control of EBV-associated malignancies and lymphoproliferative diseases, some of which have been successfully treated by adoptive T cell therapy. Therefore, many methods have been used to measure EBV-specific cellular immune responses. Previous studies have mainly used autologous EBV-transformed B-lymphoblastoid cell lines (B-LCLs), recombinant viral vectors transfected or peptide pulsed dendritic cells (DCs) as stimulators of CD8^+^ and CD4^+^ T lymphocytes. In the present study, we used an interferon-γ (IFN-γ) enzyme-linked immunospot (ELISPOT) assay by using isolated CD8^+^ and CD4^+^ T cells stimulated with mRNA-transfected DCs. The frequency of latent membrane protein 1 (LMP1)-specific IFN-γ producing CD4^+^ T cells was significantly higher than that of LMP2a. The frequency of IFN-γ producing CD4^+^ T cells was significantly correlated with that of CD8^+^ T cells in LMP1-specific immune responses (r = 0.7187, Pc < 0.0001). To determine whether there were changes in LMP1- or LMP2a-specific immune responses, subsequent peripheral blood mononuclear cells (PBMCs) samples were analyzed. Significant changes were observed in 5 of the 10 donors examined, and CD4^+^ T cell responses showed more significant changes than CD8^+^ T cell responses. CD8^+^ and CD4^+^ T cells from EBV-seropositive donors secreted only the Th1 cytokines IFN-γ, TNF-α, and IL-2, while Th2 (IL-4) and Th17 (IL-17a) cytokines were not detected. CD4^+^ T cells secreted significantly higher cytokine levels than did CD8^+^ T cells. Analysis of EBV-specific T cell responses using autologous DCs transfected with mRNA might provide a comprehensive tool for monitoring EBV infection and new insights into the pathogenesis of EBV-associated diseases.

## Introduction

Epstein-Barr virus (EBV) is a β-lymphotrophic γ-herpes virus that infects more than 90% of the world’s population [1, 2]. EBV is associated with a number of malignancies such as Hodgkin’s lymphoma (HL), Burkitt’s lymphoma, post-transplant lymphoproliferative disorder (PTLD), natural killer (NK)/T-cell lymphoma, and several lymphoepithelioma-like carcinomas, including nasopharyngeal carcinoma (NPC) and gastric carcinomas [2–6]. Recent studies have suggested that EBV also contributes to several autoimmune diseases, including multiple sclerosis, systemic lupus erythematosus, rheumatoid arthritis, and primary Sjögren syndrome [2, 7–9].

Healthy individuals are relatively unlikely to suffer life-threatening disorders induced by EBV, because EBV-specific T cells play a key role in controlling viral replication and latency establishment during primary infection [3, 10, 11]. However, further studies regarding the accurate measurement of EBV-specific T cell responses in immunocompromised patients are necessary. Functional studies on T cell reactivity to EBV antigens have been performed using proliferation [12, 13] and cytotoxicity assays [3, 14, 15]. EBV-specific T cell responses have also been detected by measuring cytokine expression with methods such as intracellular cytokine staining (ICS) [2, 16–19], enzyme-linked immunosorbent assay (ELISA) [3, 12, 14], and ELISPOT assay [11–13, 15, 20, 21]. The ELISPOT assay is a very sensitive technique for measuring the frequency of cytokine-secreting cells at the single-cell level.

The distribution of EBV-specific T cell responses has also been determined by ELISPOT assay [2, 11]. These assays primarily use Epstein–Barr nuclear antigen 1 (EBNA1), EBNA3 family, LMP1, and LMP2 as EBV latent antigens. B-LCLs, DCs [15], and PBMCs pulsed with peptides [2, 3, 12–14, 20] or transduced with recombinant viral vectors [15, 22, 23] have been used for antigen presentation. These studies mainly determined CD8^+^ T cell responses using peptides [20], as well as CD4^+^ T cell responses using vaccinia virus-transduced cells [15, 22, 23] or peptide mixtures [2, 11, 13, 14].

EBV infects primarily human B and epithelial cells, but it has been reported to be sensed by dendritic cells (DCs) during primary infection [24]. EBV DNA triggers TLR9-mediated recognition of the virus in plasmacytoid DCs, B cells, and monocytes [25–27]. TLR2 and 3 have been implicated in EBV recognition by macrophages and conventional DCs [28–30]. These DC populations seem to play significant roles during primary EBV infection along these lines plasmacytoid dendritic cells (pDCs) are potent sources of type 1 interferons (IFN-α and β) [31]. These activated DCs are thought to contribute to innate restriction of EBV infection and initiate EBV-specific adaptive immune responses via cross-priming. Indeed with the advent of mice with reconstituted human immune system compartments, which recapitulate primary EBV infection and EBV-associated lymphomagenesis, it becomes feasible to define DC populations that are involved in the priming of protective immune responses in vivo [32]. In this preclinical model, CD4^+^ and CD8^+^ T cells mediate immune control over EBV infection and B-cell lymphoma development and protective EBV-specific CD4^+^ T cells can be primed with vaccine candidates [33–35].

EBV is now considered etiologic factor in multiple types of cancer that primarily develop in lymphocytes and epithelial cells. A third major type of latency in EBV-associated malignancies is Latency II, in which LMP1, LMP2a and LMP2b proteins are expressed in addition to the Latency I genes such as EBNA1. Because LMPs have been main target antigens used for adaptive T cell therapy, the accurate measurement of T cell responses specific for LMPs could provide helpful information.

The methods to introduce RNA into DC by electroporation have been demonstrated to induce higher efficiency, allowing easy access of RNA-encoded Ags into the cytoplasmatic translation machinery upon entry into the cells and has been reported as an efficient means for whole antigen-loading of T cell-stimulating cells [36, 37]. The possibility to measure T-cell responses against whole antigens using clonal mRNA as an antigen format have been also demonstrated in ELISPOT assays. The detection of peptide-specific T cells with mRNA-electroporated APC was as sensitive as with APC exogenously loaded with an excess of peptides. Another advantage of using RNA over peptides as Ag is that RNA encodes multiple epitopes for many HLA alleles, and, therefore, extends the scope to potent T cell epitopes which have not yet been identified. An additional advantage of mRNA as opposed to plasmid DNA concerns the observation that plasmid transfection regularly induced non-specific background reactivity most likely due to bacterial contaminants (e.g., LPS) often found in plasmid preparations [37, 38]. It can be handled at a rather low laboratory safety level to compared with viral vector system to transducer DCs and does not induce non-specific background reactivity because of the absence of highly immunogenic vector sequences potentially masking the detection of low-frequency T-cell responses [39].

In the present study, we established an IFN-γ ELISPOT assay using DCs transfected with LMP1 or LMP2a mRNA to define EBV-specific cellular immunity in Korean healthy donors. Two advantages of this approach are that is allows determination of both CD8^+^ and CD4^+^ responses to epitopes, which are naturally processed from full-length LMP1 or LMP2a and presented by HLA class I and class II, and that it is applicable to individuals independent of HLA type.

## Material and Methods

### Isolation of CD14^+^, CD8^+^, or CD4^+^ Cells

Candidate donors were serologically tested to confirm remote EBV infection by enzyme immunoassays (EIAs) using VCA IgG, VCA IgM and EBNA-1 IgG antibodies (Trinity-Biotech, Ireland). In this study, 23 EBV seropositives and 4 seronegatives were subjected. Analyzed expression of EBNA-3C genes by quantitative polymerase chain reaction (qPCR) using the DNA purified from PBMCs, there was not detected in donors.

DCs were cultured in RPMI supplemented with penicillin (100 U/mL), streptomycin (100 U/mL), L-glutamine (2 mM; all from Lonza, Basel, Switzerland), and 10% fetal bovine serum (Life Technologies, Carlsbad, CA, USA). PBMCs were collected by leukapheresis followed by centrifugation on a Ficoll-Paque (GE Healthcare Bio-Sciences) density gradient after informed consent had been obtained from healthy individuals. CD14^+^ cells were isolated from PBMCs by positive selection using an anti-CD14 monoclonal antibody (mAb) coupled to magnetic microbeads (Macs, Miltenyi Biotec, Bergisch Gladbach, Germany) and sorting using an AutoMACS Pro (Miltenyi Biotec), as recommended by the manufacturer. CD8^+^ and CD4^+^ T cells were isolated from CD14^-^ cells by positive selection using anti-CD8 and anti-CD4 mAbs coupled to Macs and sorted as for CD14^+^.

### Ethics statement

This study was approved by the Institutional Review Board (IRB) of the Catholic University of Korea (IRB Number: MC13ICISI0083). Written informed consent was obtained from all participates involved in this study.

### Production of mRNA by In Vitro Transcription

The sequences encoding recombinant full-length LMP1, LMP2a and GFP were cloned into the pcDNA3 expression vector (Invitrogen, Grand Island, NY, USA). The purified plasmids (LMP1-pcDNA3, LMP2a-pcDNA3 and GFP-pcDNA3) were linearized by digestion with the restriction enzyme Sma I and purified using the Nucleospin Gel and PCR Clean-up Kit (Macherey-Nagel, Duren, Germany). mRNAs were transcribed from the linearized plasmids using an Ambion mRNA T7 Ultra Kit (Life Technologies) according to the manufacturer’s instructions.

### Synthetic Peptides

Peptide pools spanning (pepmix) full-length LMP1 (94 peptides) and LMP2a (122 peptides) were purchased from JPT Peptide Technologies, Berlin, Germany. Peptides were 15 amino acids in length with an 11-amino-acid overlap. The pepmix of each EBV protein were prepared according to the manufacturer`s instructions. Briefly, lymphilized peptides (25ug/vial) were dissolved in 80μL puro DMSO and diluted with 2420μL PBS. A final concentration of 1μg/mL for each pepmix was used in experiments.

### Generation of DCs and mRNA Electroporation

DCs were generated from isolated CD14^+^ cells after 7 days of culture in complete RPMI medium supplemented with 100 ng/mL IL-4 (Gentaur, Brussels, Belgium) and 100 ng/mL GM-CSF (Gentaur). Cultures were supplemented again on day 3 with IL-4 and GM-CSF. Maturation of immature DCs (iDCs) at 1 × 10^6^ cells/mL was at 37°C and 5% CO_2_ for 16–18 h in complete medium supplemented with 100 ng/mL IL-4, 100 ng/mL GM-CSF, 20 ng/mL TNF-α (Endogen, Rockford, IL, USA), 10 ng/mL IL-6 (R&D Systems, Minneapolis, MN, USA), and 10 ng/mL IL-1β (R&D Systems). iDCs and mature DCs (mDCs) were transfected with 20 μg mRNA using a BTX square-wave electroporator (Harvard Apparatus, Holliston, MA, USA) with a single pulse at 300 V for 500 μs. Maturation of the electroporated iDCs was performed as described above.

### Analysis of phenotype to DCs by flow cytometry

The expressions of HLA, costimulatory molecules and GFP to DCs were analyzed by flow cytometry (FACS Caliber, BD Biosciences). The cells were washed once in PBS and stained for 30min with PE-conjugated anti-HLA-A, -B, -C, anti-4-1BBL, anti-CD80, anti-CD83, and anti-CD86, FITC-conjugated anti-HLA-DR, -DP, -DQ (BD Biosciences, USA). And the cells were washed followed fixed using 1% paraformaldehyde solution. Approximately more than 10,000 cells were acquired and the data were analyzed by using FLOWJO software (Tree Star, USA).

### IFN-γ ELISPOT Assay

The ELISPOT assay was performed as described in the literature with modifications [40]. Briefly, a multi-screen 96-well plate (ELISPOT Human IFN-γ ELISPOT set, BD, Franklin Lakes, NJ, USA) was coated with IFN-γ capture antibody and incubated overnight at 4°C. The wells were washed 3 times with phosphate-buffered saline (PBS) and then blocked with RPMI 1640 for at least 2 h, followed by removal of the blocking medium and washing with PBS. Autologous CD8^+^ and CD4^+^ T lymphocytes (10^6^ cells) were serially diluted in complete RPMI and 100 μL of each concentration was transferred into the wells, followed by the addition of 200 μL transfected DCs (10^5^ cells) in complete RPMI. After 24 h, the cells were removed and the plates were washed 4 times with PBS/0.05% Tween 20. Biotinylated detection antibody for IFN-γ was added and incubated for 2 h at 37°C. After 4 washes with PBS/0.05% Tween 20, avidin-horseradish peroxidase (HRP) was added and plates were incubated for 1 h at 37°C. The plates were washed 4 times with PBS/0.05% Tween 20, followed by 2 washes with PBS and the addition of 100 μL AEC substrate (BD) per well. After 8 minutes the reaction was stopped by washing with deionized water, and the plates were dried overnight prior to membrane removal. The spot number was determined in an AID Elispot Reader System (AID Diagnostika GmbH, Strassberg, Germany).

### ELISA for Cytokines

Supernatants from wells containing DCs and T cells for the ELISA in 96 well U-bottom plate were also used to measure cytokines (IL-2, IFN-γ, TNF-α, IL-4, and IL-17a) by ELISA (BioLegend, USA). Briefly, ELISA plates were coated with each cytokine capture antibody (4 μg/mL) overnight at 4°C and then the plates were blocked with 200 μL of 1:5 diluted 5× Assay Diluent A added to each well for 1 h at room temperature. The plates were then washed and incubated with collected supernatants (100 μL/well) for 2 h at room temperature, followed by washing and incubating with detection antibody (100 μL/well) for each cytokine for 1 h at room temperature. After washing, the plates received 100 μL avidin-HRP solution per well and were incubated for 30 min at room temperature. The plates were then washed and incubated with 100 μL/well TMB substrate solution for 15 min at room temperature in the dark, after which the reaction was quenched with 100 μL of Stop solution (2N H_2_SO_4_). The antibody levels were measured in a Multilabel Counter System (PerKin Elmer, Finland).

### Statistical Analysis

Data were evaluated using GraphPad Prism version 5.0. Comparisons between experimental groups were performed using the spearman test and the Mann–Whitney test. The inter-assay cultured ELISPOT variability was calculated as the %CV of the mean from duplicate wells from each assay performed on different days. The corrected P (Pc) values were adjusted by using Bonferroni`s correction for multiple comparisons to correlation. All *p*-values reported are two-sided and significance was set at *p* < 0.05.

## Results

### Optimization of IFN-γ ELISPOT assay

**Fig 1:**
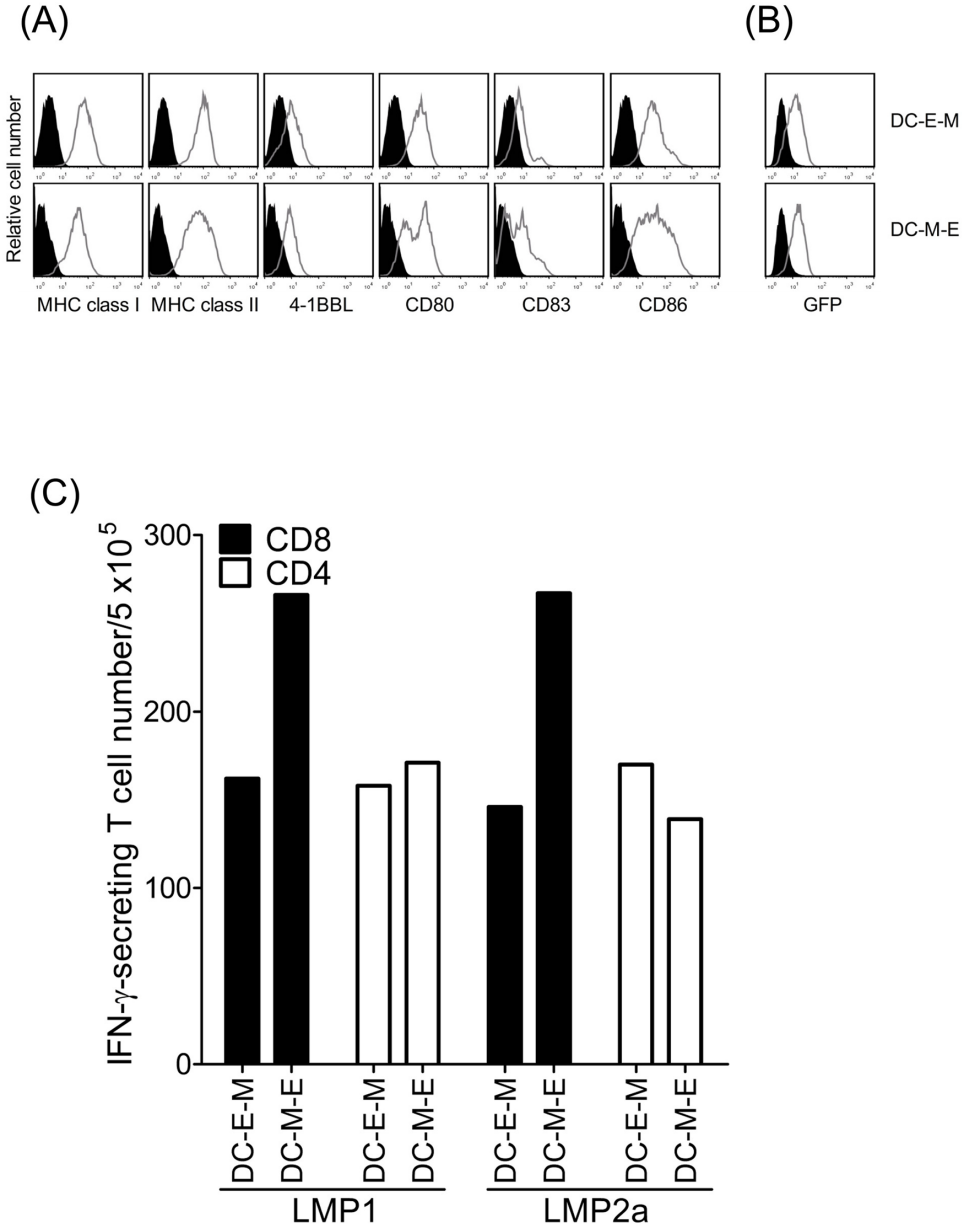
The immunophenotype and stimulatory capacity of GFP mRNA-transfected DCs before (DC-E-M) or after (DC-M-E) maturation. (A, B) Comparison of expression levels of HLA class I and II molecules, 4-1BBL, CD80, CD83, CD86, and GFP in DCs by flow cytometric analysis using specific mAbs. Histograms compare binding of specific mAbs (gray) and control (black). (C) Stimulatory capacity of DCs transfected by electroporation before or after a 24-h maturation period with 20 μg LMP1 or LMP2a mRNA. This experiments were repeated in #18 donor.

We compared the effect of mRNA electroporation in immature and mature DCs using GFP mRNA (Fig 1A and 1B). The cell viability and expression of HLA class I and II molecules, costimulatory molecules (4-1BBL, CD80, CD83, and CD86), and GFP were not different between mRNA-electroporated iDCs (DC-E-M) and mDCs (DC-M-E). However, the recovery rate of DC-M-E was higher than that of DC-E-M (53.3% ± 4.7% vs. 44% ± 11.4%, respectively; n = 3). In donor #18 showing positive T cell response, transfection of mDCs resulted in greater numbers of IFN-γ-secreting CD8^+^ T cells (266 and 267 per 5 × 10^5^ cells, respectively) than iDCs (166 and 146 per 5 × 10^5^ cells, respectively), for both LMP1 and LMP2a. However, in CD4^+^ T cells, while LMP1-transfected mDCs also stimulated greater numbers of IFN-γ-secreting cells than the corresponding iDCs (171 vs. 158 per 5 × 10^5^ cells, respectively), LMP2a-transfected iDCs stimulated a greater number than the corresponding mDCs (139 vs. 170 per 5 × 10^5^ cells, respectively) (Fig 1C). This experiments were repeated in #18 donor. As a result, we performed mRNA transfection on mDCs in subsequent experiments due to the more efficient antigen presentation.

### Comparison between mRNA transfection and pulsing with pepmix

**Fig 2:**
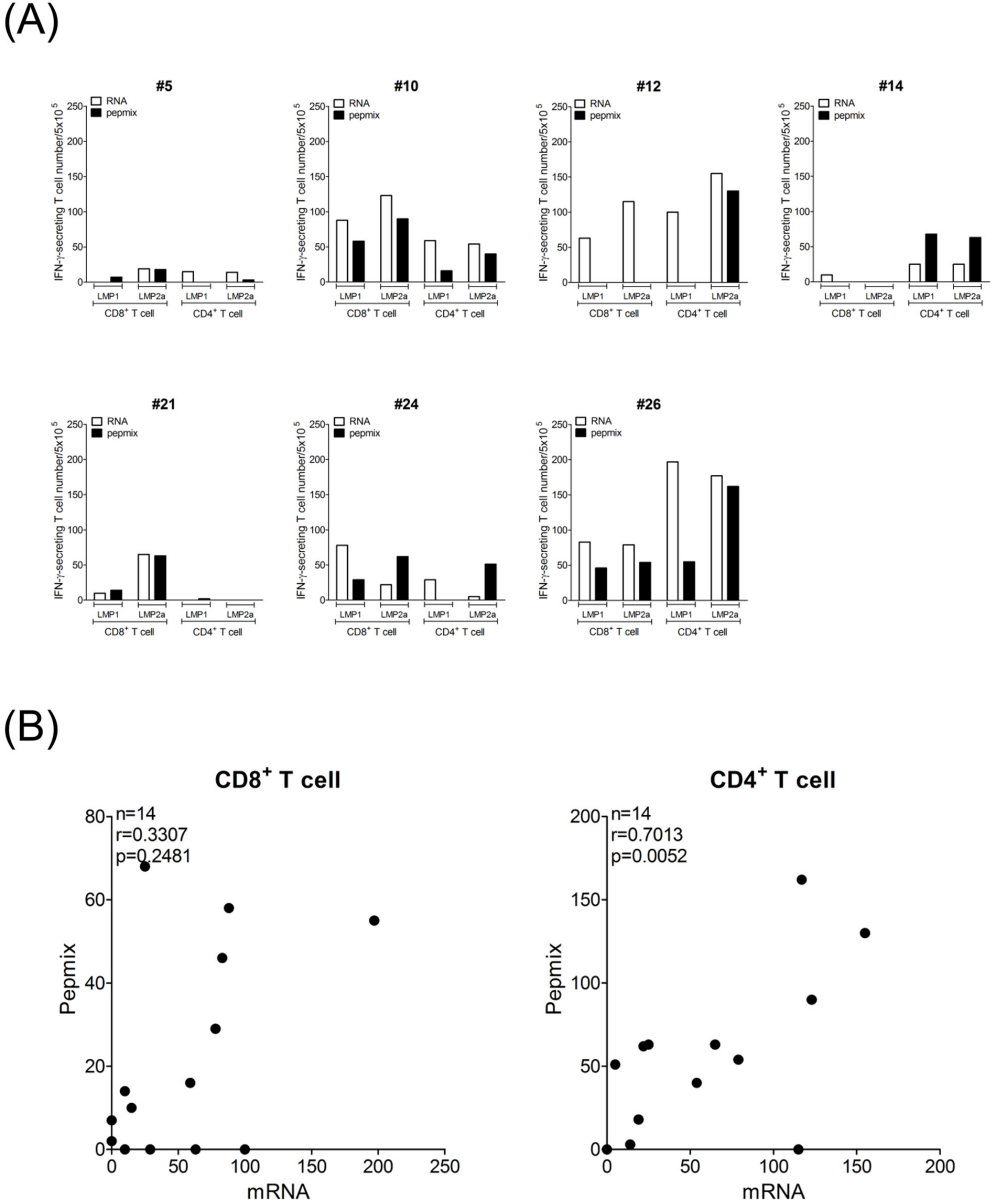
Comparison of IFN-γ ELISPOT responses specific to LMP1 and to LMP2a between DCs transfected with mRNA and pulsed with pepmix. (A) IFN-γ ELISPOT responses using mRNA and pepmix were measured in 7 donors. (B) Correlation of LMP1- and LMP2a-specific IFN-γ ELISPOT responses between mRNA and pepmix was analyzed in CD8+ and in CD4+ T cells of 7 donors.

To address the rationale and advantages of using the mRNA transfection system with regards to other stimulation approaches, LMP1 and LMP2a-specific IFN-γ ELISPOT assays using mRNA-transfected DCs has been compared with that using DCs pulsed with pepmix because these two methods are not restricted by particular HLA alleles. Among total 28 IFN-γ ELISPOT responses from CD8+ and CD4+ T cells specific to LMP1 and to LMP2a in 7 donors, mRNA-transfected DCs showed higher frequencies than pepmix in 22 responses (Fig 2A). CD4+ T cell frequencies showed significant correlation between mRNA transfection system and pepmix (r = 0.7013, p = 0.0052), but CD8+ T cell frequencies was not correlated between two methods (Fig 2B). These results suggest that mRNA transfection system is feasible to measure T cell responses to whole antigen.

### Measurement of immune responses to EBV

**Fig 3:**
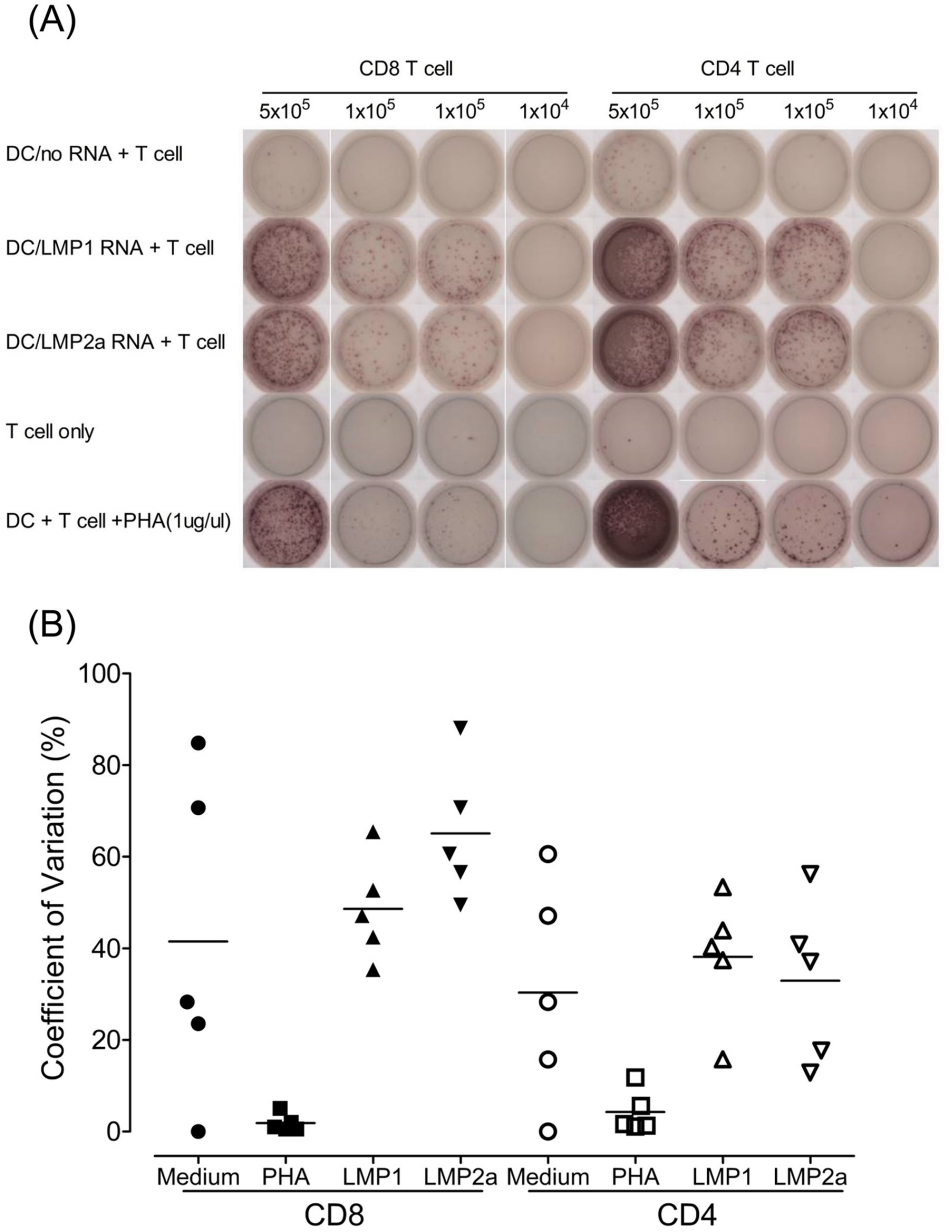
Measurement of immune responses to EBV antigen by IFN-γ ELISPOT assay in healthy donors. (A) Production of IFN-γ by CD8^+^ and CD4^+^ T cells against various stimulators such as DCs, LMP1- and LMP2a mRNA-transfected DCs, or phytohemagglutinin (PHA; 1 μg/μL). (B) Reproducibility of the ELISPOT assay. Inter-assay variations were evaluated in ELISPOT assays using CD8^+^ and CD4^+^ T cells from five EBV-seropositive healthy donors and LMP1 and LMP2a mRNA-transfected DCs, with PHA as the positive control. For the inter-assay variability, each symbol represents the mean of duplicate wells from each assay performed on two different days.

Fig 3A shows representative IFN-γ ELISPOT results for the detection of LMP1- or LMP2a-specific CD8^+^ and CD4^+^ T cells. The most intense IFN-γ spots were obtained in wells containing LMP1 or LMP2a mRNA-transfected DCs plus T cells and in positive control wells containing 1 μg/μL PHA-stimulated DCs plus T cells. Negative control wells with no RNA transfected DCs plus T cells and T cells alone were shown background levels of spot. Multiple wells containing 5 × 10^5^, 1 × 10^5^, and 1 × 10^4^ T cells were used for measurement within the linear range of the assay.

To confirm the reproducibility of the ELISPOT assays, the same donors (n = 5) were examined on two different days by a single operator (Fig 3A). The mean inter-assay CV, describing the variation among three assays performed on different days, ranged from 1.8% to 65.1% (Fig 3B). The mean and standard deviation (SD) of the differences between total 20 inter-assays were 18.1 and 19.6 spots per 5 × 10^5^ cells.

### LMP1 and LMP2a-specific T cell frequencies in healthy Korean donors

**Fig 4:**
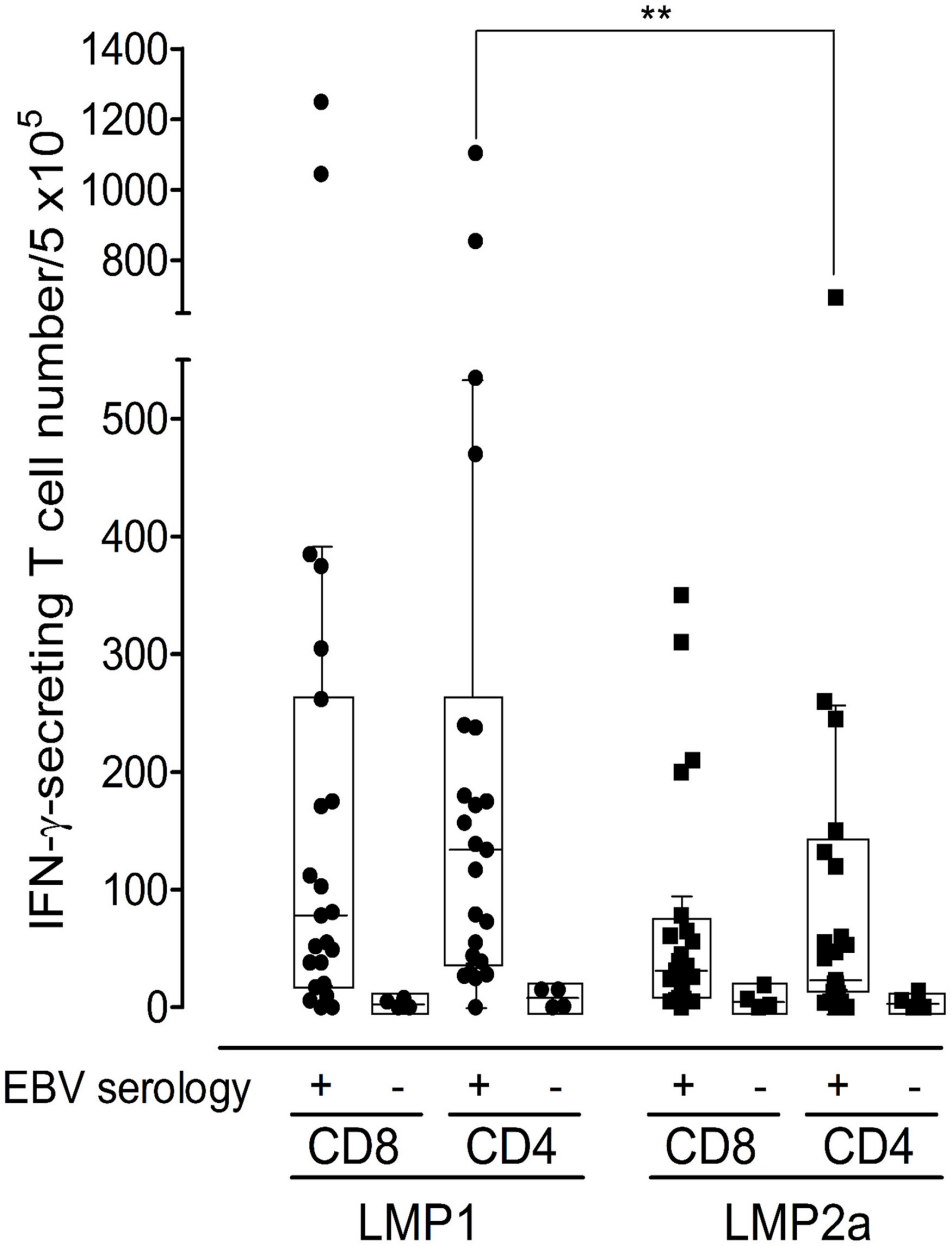
Frequency of LMP1- or LMP2a-specific CD8^+^ and CD4^+^ T cell responses of healthy Korean donors. Monocyte-derived DCs isolated from peripheral blood mononuclear cells (PBMCs) from EBV-seropositive (n = 23) and EBV-seronegative (n = 4) healthy donors were evaluated in response to mRNAs for latent (LMP1 and LMP2a) EBV antigens. Results are shown as IFN-γ secreting T cell number/5 × 10^5^ for IFN-γ ELISPOT responses. EBV-specific CD4^+^ T cell responses determined by the LMP1 ELISPOT assay are significantly higher than those detected by the LMP2a ELISPOT assay (p = 0.0067, two-tailed Mann-Whitney test). Box and whisker plots (Tukey’s test; 25% ~ 75%) indicate median (middle line in the box) of EBV-specific T cell responses in individuals.

The frequencies of LMP1- or LMP2a-specific IFN-γ producing cells in CD8^+^ and CD4^+^ T cells in response to full-length-antigen mRNA were measured in 27 healthy Korean donors, including 4 EBV-seronegative donors (Fig 4). In EBV-seropositive donors (n = 23), the median frequency of 134 (interquartile range (IQR) 39–238) for the IFN-γ-secreting CD4^+^ T cells (per 5 × 10^5^) in response to LMP1 was significantly higher than that of LMP2a, which was 23 (IQR 12–120) (*p* < 0.01, two-tailed Mann-Whitney test). In addition, the frequency of CD8^+^ T cells in response to LMP1, 78 (IQR 20–262), was higher than that of LMP2a, 31 (IQR 7–65), although no significant difference was found. CD4^+^ T cell responses were higher than CD8^+^ T cell responses for LMP1 and similar in both CD8^+^ and CD4^+^ T cells for LMP2a. Significant differences between CD4^+^ and CD8^+^ T cell frequencies specific for LMP1 or LMP2a were not observed. In 4 EBV-seronegative donors, the median EBV-specific T cell number per 5 × 10^5^ cells detected by ELISPOT was 3.5 for both CD8^+^ T cells (IQR 0–7.8) and CD4^+^ T cells (IQR 0–14.8), consistent with non-existing EBV immunity and demonstrating the assay specificity.

**Fig 5:**
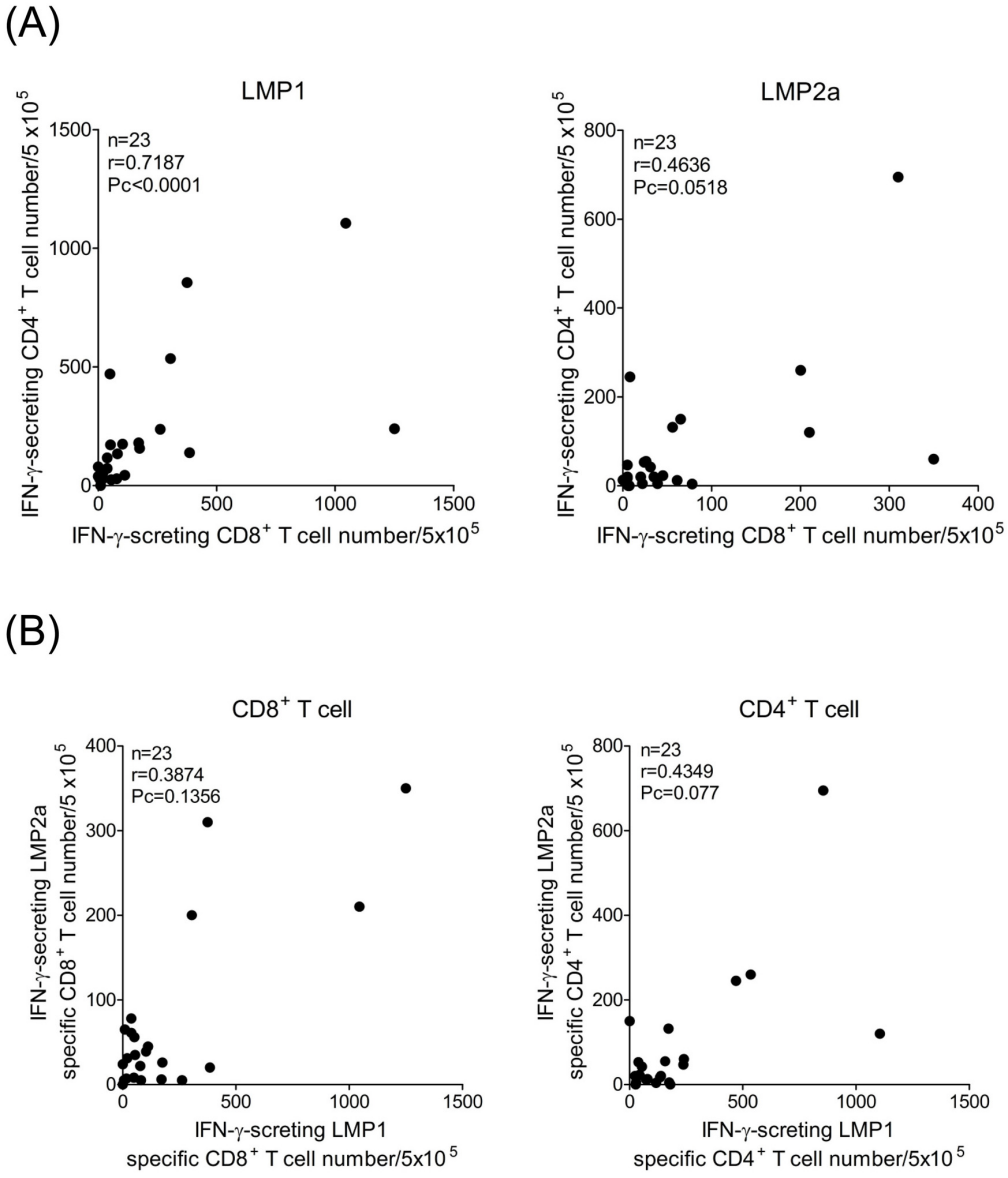
Correlations of estimates of LMP1- or LMP2a-specific T cell responses obtained using IFN-γ ELISPOT assay. (A) Correlation between LMP1- or LMP2a-specific immune response from CD8^+^ T cells expressed on the x-axis and CD4^+^ T cells expressed on the y-axis. A significant correlation was observed for LMP1 between results obtained from CD8^+^ and CD4^+^ T cell responses (n = 23, r = 0.7187, Pc < 0.0001). (B) Correlation between LMP1-specific CD8^+^ and CD4^+^ T cell immune responses on the x-axis and LMP2a-specific CD8^+^ and CD4^+^ T cell responses on the y-axis.

For the LMP1- and LMP2a-specific immune responses investigated in CD8^+^ and CD4^+^ T cells, only the values of the assay were analyzed. In Fig 5A, LMP1-specific immune responses were shown a high correlation between CD8^+^ and CD4^+^ T cells (n = 23, r = 0.7187, Pc < 0.0001). However, LMP2a-specific CD8^+^ and CD4^+^ T cell responses did not show a significant correlation. LMP1-specific immune responses were not correlated with that of LMP2a (Fig 5B).

### LMP1- and LMP2a-specific T cell frequencies in subsequent PBMC samples

**Fig 6:**
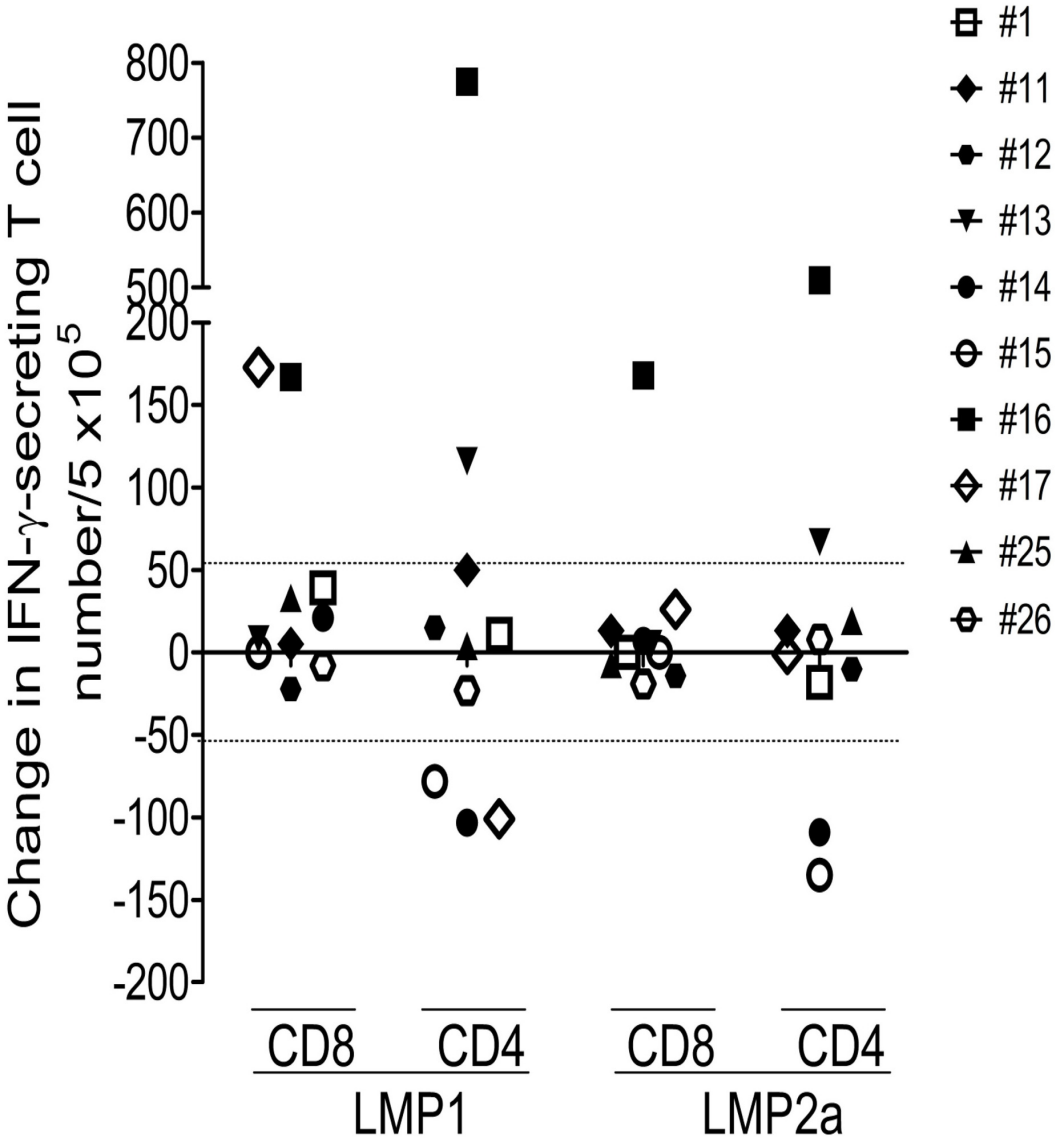
Analysis of LMP1- or LMP2a-specific immune responses by IFN-γ ELISPOT assays in a subsequent T cells (3–15 months apart). LMP1 or LMP2a proteins were obtained from ten EBV-seropositive healthy donors. Result is shown as variation in IFN-γ-secreting T cell number per 5 × 10^5^ cells.

To determine whether LMP1- or LMP2a-specific immune responses changed over time, subsequent PBMC samples were collected from 10 donors during a period of 3–15 months (Fig 6). Because the mean + 2 × SD of the differences between inter-assays were 57.3, it was regarded as a significant change that if the IFN-γ secreting T cell frequency increased or decreased by more than 58 spots per 5 × 10^5^ cells. LMP1-specific CD8^+^ T cell responses increased in 2 donors, and CD4^+^ T cell responses increased in 2 donors and decreased in 3 donors. LMP2a-specific CD8^+^ T cell responses increased in 1 donor, and CD4^+^ T cell responses increased in 2 donors and decreased in 2 donors. There were significant changes in 5 donors, and CD4^+^ T cell responses (5 donors) showed more significant changes than CD8^+^ T cell responses (2 donors). Decreases in 3 donors were observed only in LMP1- or LMP2a-specific CD4^+^ T cell responses.

### Correlation of secreted cytokine levels with ELISPOT

**Fig 7:**
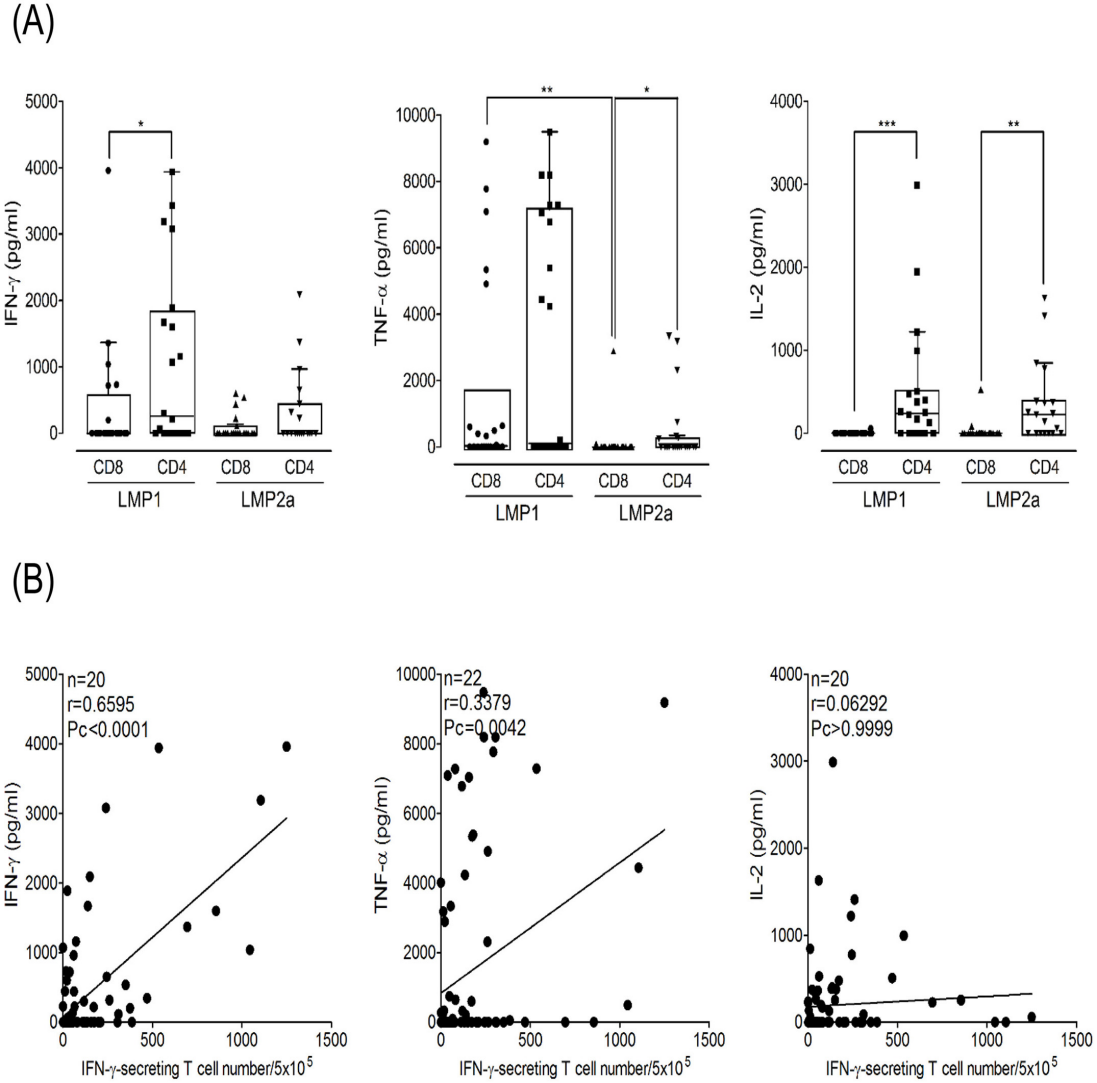
Frequency of LMP1- or LMP2a-specific immune responses by ELISA and comparison of IFN-γ ELISPOT assay. (A) Frequencies of IFN-r (n = 20), TNF-a (n = 22) and IL-2 (n = 20) levels were investigated with supernatant which were secreted in CD8^+^ and CD4^+^ T cells stimulated with LMP1 or LMP2a mRNA-transfected DCs. Box and whisker plots (Tukey’s test; 25% ~ 75%) indicate median (middle line in the box) of secreted cytokine level in individuals. (B) Comparison of estimates of cellular immune responses obtained using ELISPOT and cytokine ELISA. The results of the IFN-γ ELISPOT assays correlated well with those from IFN-γ ELISA (n = 20, r = 0.6595, Pc < 0.0001). (*: p < 0.05, **: p < 0.01, ***: p < 0.0001)

Concentrations of IFN-γ, TNF-α, IL-2, IL-4, and IL-17a secreted from LMP1- or LMP2a-specific CD8^+^ and CD4^+^ T cells were measured using a cytokine ELISA (Fig 7). T cells from EBV-seropositive donors secreted only the Th1 cytokines IFN-γ, TNF-α, and IL-2, while Th2 (IL-4) and Th17 (IL-17a) cytokines were not detected. The CD4^+^ T cells secreted significantly higher cytokine levels than CD8^+^ T cells.

The secretion of IFN-γ from LMP1-specific CD4^+^ T cells was higher than that of CD8^+^ T cells (*p* = 0.0426). IFN-γ level were also significantly correlated with T cell frequency by IFN-γ ELISPOT (n = 20, r = 0.6595, Pc < 0.0001). TNF-α secreted from LMP2a-specific CD4^+^ T cells was higher than that from CD8^+^ T cells (*p* = 0.0495), and that from LMP1-specific CD8^+^ T cells was higher than that from LMP2a-specific CD8^+^ T cells (*p* = 0.0029). There was a moderate correlation between TNF-α level and T cell frequency by IFN-γ ELISPOT (n = 20, r = 0.3379, Pc < 0.0042). IL-2 levels secreted from LMP1- and LMP2a-specific CD4^+^ T cells were higher than those from CD8^+^ T cells (*p* < 0.0001 and *p* = 0.0007, respectively).

## Discussion

EBV-specific T cell responses have been studied in detail, and primarily CD8^+^ and CD4^+^ T cell responses and expansion during primary infection have been clearly demonstrated [41–44]. Previous studies have mainly used autologous EBV-transformed B-LCLs as stimulators of CD8^+^ and CD4^+^ T lymphocytes [45, 46]. B-LCLs express the latent and lytic proteins of EBV, owing to the fact that a small proportion of B-LCLs (< 5%) enter the lytic phase of infection [47]. However, attempts to generate autologous B-LCLs for diseased peoples are not always successful. We performed direct comparison experiments between LMP1/2 pulsed APCs and LCLs in 2 donors (S1 Fig). LCLs showed higher background IFN-γ ELISPOT responses in both CD8+ and CD4+ T cells compared with DCs not transfected with antigen mRNAs. In donor #14 which has low T cell responses, LMP1- and LMP2a-specific T cell responses can be not measured because LCLs transfected with mRNAs showed rather lower T cell responses than mock LCLs. Although LCLs transfected with mRNAs showed higher T cell responses than mock LCLs in donor #26, DCs transfected with mRNA were more useful to measure LMP1- and LMP2a-specific T cell responses compared with LCLs transfected with mRNA because of lower background level. The LCLs express not only the latent proteins of EBV but also lytic proteins and a small proportion of cells in the LCLs enter into the lytic phase of infection. Therefore, LCL may be inappropriate for the measurement of immune response against a single antigen. In the present study, we analyzed antigen-specific CD8^+^ and CD4^+^ T cell responses to autologous DCs transfected with LMP1 and LMP2a mRNAs by IFN-γ ELISPOT and cytokine ELISA. It has been shown that mature DCs are potent stimulators of T cells *in vitro*. For clinical applications, recombinant adenoviruses are attractive vectors for the genetic modification of DCs, because high transduction rates are achieved without interfering with DC function in comparison to other viral vectors, such as vaccinia or herpes simplex virus [48, 49]. Adenoviral-transduced DCs have been used successfully in vitro to generate specific CTLs against a variety of tumor-associated antigens [15]. On the other hand, since mRNA is degraded over time, the maturation of DCs after transfection would result in a shortened duration of antigen presentation, leading to reduced T-cell stimulatory capacity. It is known that iDCs can play a role in antigen capture and processing, whereas mDCs present antigens and have increased T cell stimulatory capacity. In support of the concept of introducing mRNA into immature DCs, previous studies have used immature DCs for loading with mRNA either by lipofection or eletroporation [37, 50]. Based on the level of IFN-γ secretion, our results show that DCs transfected after maturation were more potent in inducing an autologous antigen-specific CD8^+^ T cell response than those transfected prior to maturation (Fig 1C). In general, the EBV-specific CD4^+^ T cell response is still poorly defined, primarily due to the small size of the CD4^+^ memory T cell compartment and the paucity of defined CD4^+^ EBV epitopes. Our results demonstrate that DCs transfected with antigen mRNAs after induction of their mature phenotype can be used to detect both CD8^+^ and CD4^+^ T cell responses *in vitro*. Because an entire protein-spanning mixture (pepmix) of overlapping peptides, presented by a variety of HLA alleles, is efficient for in vitro stimulation of T lymphocytes, they have been used as attractive alternatives for the ELISPOT and intracellular cytokine staining assays. CD8-depleted PBMCs were higher than CD4-depleted PBMCs in EBV-specific immune responses using pepmix [11].

In the cellular immune control of EBV latency I and II malignancies, the primary role of CD8^+^ and CD4^+^ T cells might be as cytotoxic T cells that are able to lyse targets presenting EBNA1 and/or LMP1 on MHC class II. Against EBV latency III, however, it is likely that CD4^+^ T cells play a pivotal role in supporting CD8^+^ T cell responses that preferentially target the EBNA3 and LMP2 proteins. LMP1 is poorly recognized by CD8^+^ T cells, but targeted frequently by CD4^+^ T cells [23]. However, LMP2 elicits robust CD8^+^ T cell responses, while CD4^+^ T cell responses are rarely seen [13, 23]. Although significant differences between CD8^+^ and CD4^+^ T cell frequencies specific for LMP1 or LMP2a were not observed, CD4^+^ T cell responses were higher than CD8^+^ T cell responses against LMP1, and similar responses were seen in CD8^+^ and CD4^+^ T against LMP2a. The hierarchy of immunodominance, which was more evident for CD4^+^ T cell responses detected by ELISPOT, can be summarized as EBNA1 > EBNA3 family antigens > LMP2 > LMP1 [11]. Our results show that the CD4^+^ T cell response to LMP1 was significantly higher than to LMP2a. However, differences between CD8^+^ and CD4^+^ T cell responses to LMP1 or LMP2a were not observed (Fig 4). Previous evidence in HIV infection has suggested that there is a positive correlation between CD4^+^ T cell proliferation and the frequency of CD8^+^ T cells [51]. The current study demonstrates a significant correlation between CD8^+^ and CD4^+^ T cell immune responses to LMP1 (Fig 5); however, there was no correlation to LMP2a. Most of the studies involving EBV infection have shown that T cell responses to the latency antigens are predominantly Th1-polarized with a broad IFN-γ secretion [12, 22, 52, 53]. Similarly, we observed that CD4^+^ T cells from EBV-seropositive donors secreted only the Th1 cytokines IFN-γ, TNF-α, and IL-2, whereas Th2 (IL-4) and Th17 (IL-17a) cytokines were not detected (Fig 7). DCs could be responsible for this Th1 skewing because of their high production of IL-12 [54, 55].

Our study demonstrates that IFN-γ ELISPOT and cytokine ELISA of naturally processed epitopes using autologous DCs transfected with mRNA can be reliably used to measure EBV-specific T cell responses, and that the distribution of T cell responses is relatively stable in healthy EBV carriers. Furthermore, this strategy would not only facilitate investigations of EBV-specific CD4^+^ T cell responses, but would also be useful for the quantification of T cell responses to various antigens expressed at different stages of viral latency. Therefore, analyses of EBV-specific T cell responses using autologous DCs transduced with mRNA might provide comprehensive tools for monitoring EBV infection and new insights into the pathogenesis of EBV-associated diseases.

## Supporting Information

S1 Fig Comparative measurement of T cell immune response to LMP1 and LMP2a between LCL and DCs (N = 2). (DOCX)
