# Supplementary material for: Measurement of CD8+ and CD4+ T Cell Frequencies Specific for EBV LMP1 and LMP2a Using mRNA-Transfected DCs
Source: PLoS One. 2015 May 29;10(5):e0127899. doi: 10.1371/journal.pone.0127899 (PMC4449191; doi:10.1371/journal.pone.0127899)
Supplement: S1 Fig — (DOCX) [file pone.0127899.s001.docx]

**Supporting Information Captions**

**
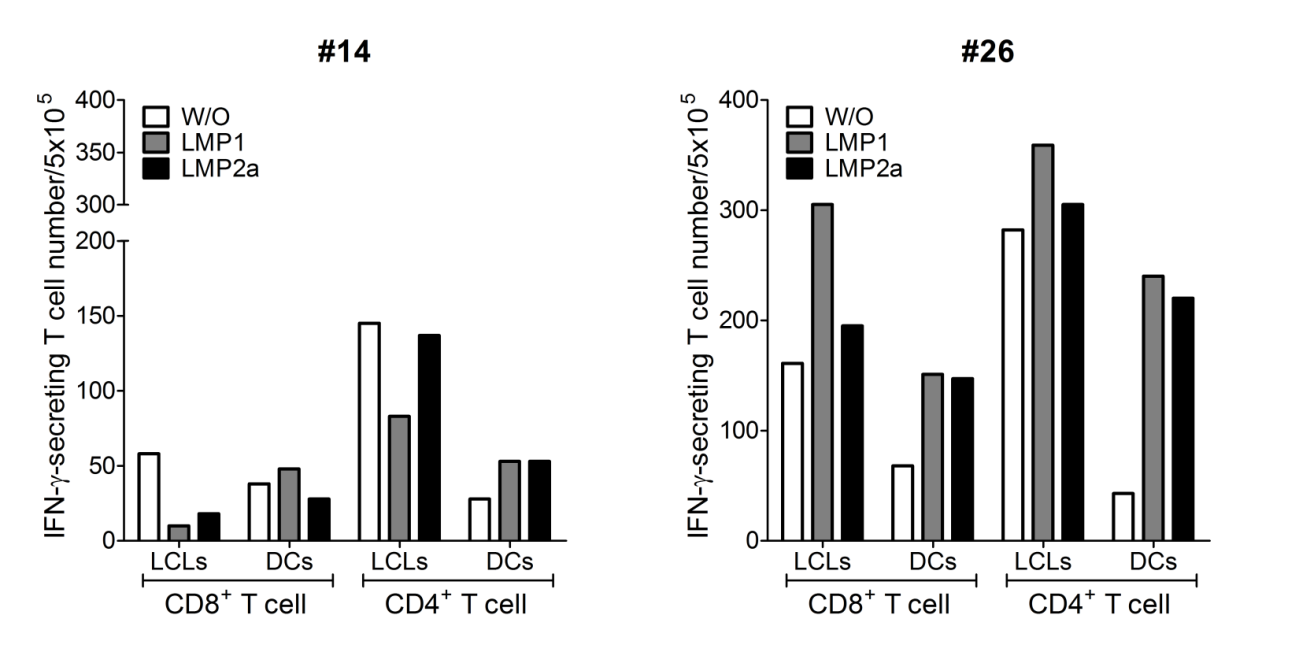
**

**S1 Fig. Comparative measurement of T cell immune response to LMP1 and LMP2a between LCL and DCs (N = 2).**
